# Low-intensity ultrasound attenuates paw edema formation and decreases vascular permeability induced by carrageenan injection in rats

**DOI:** 10.1186/s12950-020-0235-x

**Published:** 2020-02-13

**Authors:** Kil Hwan Kim, Hyeon-Woo Im, Mrigendra Bir Karmacharya, Sejong Kim, Byoung-Hyun Min, So Ra Park, Byung Hyune Choi

**Affiliations:** 1 Veterans Medical Research Institute, Veterans Health Service Medical Center, Seoul, Republic of Korea; 2 Department of Physiology and Biophysics, Inha University College of Medicine, 100 Inha-ro, Nam-gu, Incheon, 22212 Republic of Korea; 3 Department of Radiology, Perelman School of Medicine, University of Pennsylvania, Philadelphia, USA; 4 Department of Biomedical Sciences, Inha University College of Medicine, 100 Inha-ro, Nam-gu, Incheon, 22212 Republic of Korea; 5 Department of Orthopaedic Surgery, School of Medicine, Ajou University, Suwon, Republic of Korea

**Keywords:** Mechanical stimulation, Low-intensity ultrasound (LIUS), Paw edema, Vascular permeability, Inflammation

## Abstract

**Background:**

Therapeutic potential of low-intensity ultrasound (LIUS) has become evident in various musculoskeletal diseases. We have previously shown that LIUS has an inhibitory effect on local edema in various diseases including the arthritis and brain injury. In this study, we examined whether LIUS can attenuate paw edema formation vis-à-vis vascular permeability and inflammation in rats induced by carrageenan. LIUS with a frequency of 1 MHz and the intensities of 50, 100, or 200 mW/cm^2^ were exposed on rat paws for 10 min immediately after carrageenan injection.

**Results:**

Carrageenan injection induced paw edema which was peaked at 6 h and gradually decreased nearly to the initial baseline value after 72 h. LIUS showed a significant reduction of paw edema formation at 2 and 6 h at all intensities tested. The highest reduction was observed at the intensity of 50 mW/cm^2^. Histological analyses confirmed that LIUS clearly decreased the carrageenan-induced swelling of interstitial space under the paw skin and infiltration of polymorphonuclear leukocytes. Moreover, Evans Blue extravasation analyses exhibited a significant decreases of vascular permeability by LIUS. Finally, immunohistochemical staining showed that expression of pro-inflammatory proteins, namely, inducible nitric oxide synthase (iNOS) and cyclooxygenase-2 (COX-2) induced by carrageenan injection was reduced back to the normal level after LIUS stimulation.

**Conclusions:**

These results provide a new supporting evidence for LIUS as a therapeutic alternative for the treatment of edema in inflammatory diseases such as cellulitis.

## Background

Edema is the result of an imbalance in water filtration between the blood vessels and interstitial spaces. Edema that commonly occurs in the feet and legs, also referred to as peripheral edema, is mainly caused by venous obstruction and increased vascular permeability. Peripheral edema can also be caused by inflammation which increases transport of transcapillary fluid. In particular, acute inflammatory response which is characterized by an increase in cellular infiltration and vascular leakage leads to extravasation of fluid including plasma proteins, accumulation of white blood cells at the inflammatory site. This subsequently causes edema.

There are several mediators involved in inflammation. For example, histamine, serotonine, and prostaglandins are involved in the increased vascular leakage. Another key mediator in acute inflammation is nitric oxide (NO) which is induced in various pathological conditions by nitric oxide synthase (NOS). After the increase in vascular leakage, there is an extensive immune cell infiltration, mainly neutrophils in the acute phase [1]. Generally, diuretic or non-steroidal anti-inflammatory drugs (NSAIDs) such as indomethacin and aspirin are often used to reduce edema and inflammation [2]. However, these drugs have shown various side effects, such as diuretic resistance, hyponatremia, gastro-intestinal irritation, and ulceration in high dosage and long-term use of them [3]. Thus more effective and convenient therapeutic approaches with much higher safety are needed.

Many studies have shown that proinflammatory proteins iNOS and COX-2 play an important pathophysiological role in the development of paw edema via massive productions of NO and prostaglandin E synthase 2 (PGE2), respectively. NO is a potent vasodilator, and its involvement during an inflammatory reaction may be related to its ability to increase vascular permeability and edema formation through changes in local blood flow [4]. Moreover, NO has been known to increase the production of proinflammatory prostaglandins in in vitro and in vivo studies [5, 6]. Paw injection of arachidonic acid produced a moderate edema that was greatly reduced by the NO scavenger hemoglobin and L-NG-nitroarginine methyl ester (L-NAME), a NO synthase inhibitor. Furthermore, both edema and prostaglandin biosynthesis induced by the combination of arachidonic acid and a NO donor 3-morpholinosydnonimine (SIN-1) were suppressed by hemoglobin but unaffected by methylene blue, an inhibitor of the soluble guanylate cyclase. These data showed that NO is involved in edema and prostaglandin biosynthesis.

Recent accumulating studies have reported that the mechanical modalities, such as ultrasound (US) and electrical stimulation have therapeutic effects in various diseases [7–10]. In particular, US at the frequency of 3 to 10 MHz has been used in general in clinical applications, mainly for diagnostic and therapeutic purposes. Especially, LIUS with an intensity less than 1 W/cm^2^ was proven to be useful in treating edema in many diseases. For instance, LIUS has been shown to attenuate brain edema induced by a traumatic injury in rats [11]; LIUS showed anti-inflammatory effects with a reduced synovial edema in a rabbit osteoarthritis model [12]; and LIUS reduced knee circumference and infiltration of inflammatory cells in arthritis in rats [13]. We have also demonstrated that LIUS inhibits the swelling of erythrocyte induced by gramicidin D [14] and the cytotoxic brain edema formation caused by water intoxication [15].

In the current study, we investigated whether LIUS can affect acute inflammatory swelling by regulating the vascular permeability and inflammatory mediators in a rat paw edema model. We employed the carrageenan-induced rat paw edema model, because it is a widely used model to determine paw edema formation and anti-inflammatory activity, and has been fully characterized previously [16, 17]. Our present study demonstrates that LIUS stimulation significantly inhibited paw edema formation and vascular permeability in carrageenan-injected rats.

## Results

### Effects of LIUS on paw edema formation

**Fig. 1:**
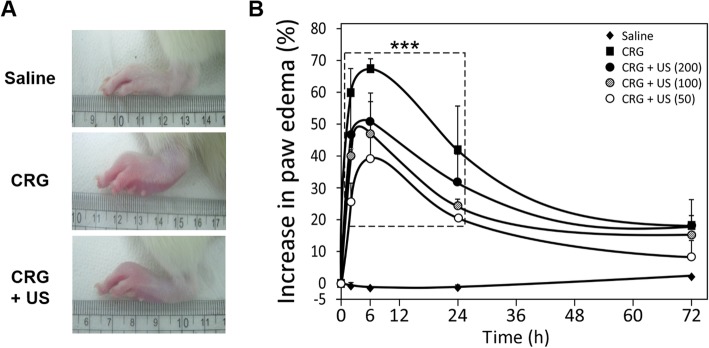
The LIUS effect on paw edema in rats. **a** Representative gross photographs displaying changes of the carrageenan-induced edema in the rat paw. Rats were injected with 1% carrageenan (100 μL) in subplantar to induce acute paw edema. Comparative photographs were obtained at 6 h following carrageenan injection in the left hind paw in the saline, carrageenan (CRG) and 100 mW/cm^2^ of LIUS stimulation (CRG + US) groups. **b** Time-course of edema formation after carrageenan injection and LIUS stimulation as 50, 100 and 200 mW/cm^2^ for 10 min. The paw volumes were measured using a plethysmometer prior to saline or carrageenan injection, and at 2, 6, 24 and 72 h after injection (*n* = 5 per group). Data are reported as means ± S.E. *P < 0.05, ** *P* < 0.01, and *** *P* < 0.001 compared to the CRG group

To investigate whether LIUS stimulation has a therapeutic effect on inflammatory edema, we first examined the effect of LIUS on paw edema induced by carrageenan in rats. The carrageenan-induced paw edema model is one of the widely used models for screening the efficacy of anti-inflammatory drugs and paw edema [18]. As shown in Fig.1a, at 6 h after subcutaneous administration of 1% carrageenan in paw, rats showed a noticeable difference in gross morphology such as increased redness and inflamed paw, resulting in paw edema. When stimulated with LIUS (CRG + US), however, the extent of the swollen paw was decreased overall. Consistent with this, quantitative analysis of the measured paw edema volume also indicated a decrease in the edema formation by post-injection particularly after 2 and 6 h (Fig. 1b). LIUS at all tested intensities of 50, 100, and 200 mW/cm^2^ showed efficient inhibition of the paw edema formation as compared to the carrageenan-injected group (CRG). Especially, LIUS with 50 and 100 mW/cm^2^ (CRG + US) showed a significant decrease in paw edema volume to 39.7 ± 6.0% and 47.1 ± 9.9%, respectively compared with the CRG group (67.4 ± 3.0%) post-injection after 6 h. The paw edema almost returned to normal after 72 h of carrageenan injection regardless of the LIUS stimulation.

### Histopathological examination in the carrageenan-injected rats

**Fig. 2:**
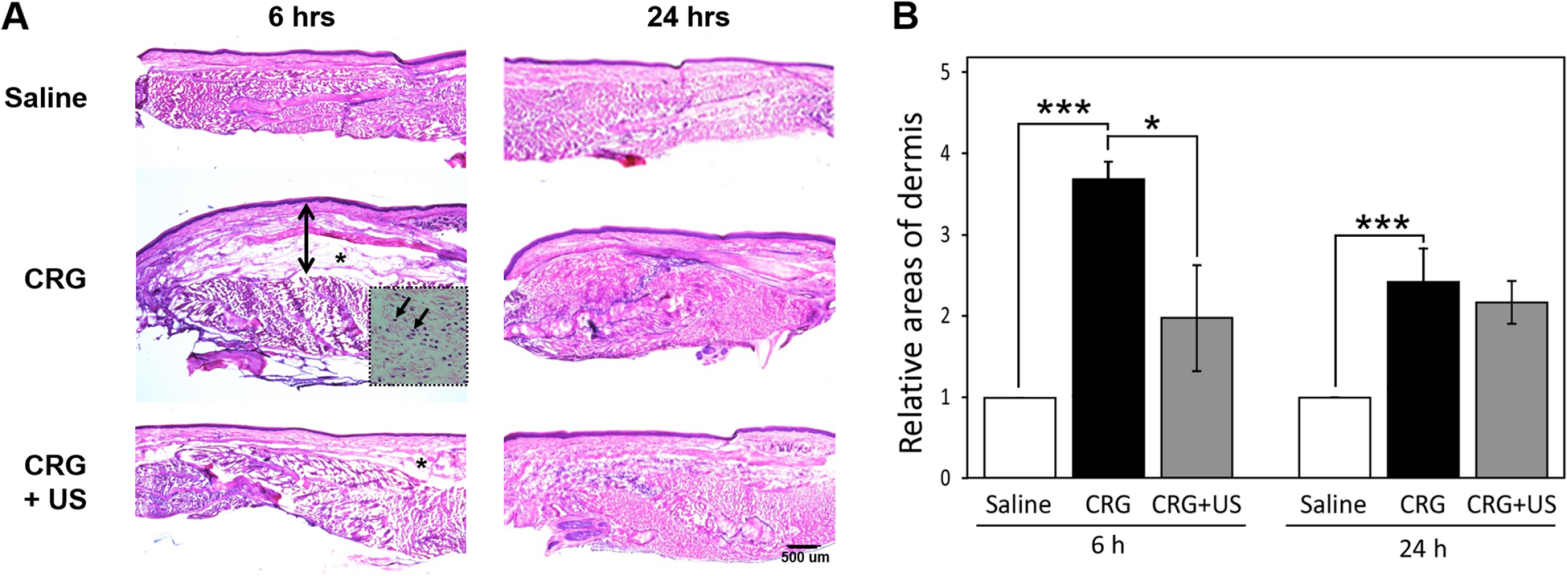
Histological analysis of paw edema. **a** Tissue sections from the hind paw were stained with H&E for histopathologic examination at 6 and 24 h in the saline (control), carrageenan-injected (CRG) and LIUS-stimulated (CRG + US) groups. The up down arrow indicates the diffused dermal layer and the asterisk shows interstitial edema in the CRG and CRG + US groups. The polymorphonuclear leukocytes are presented at higher magnification in the dotted box (× 100). **b** Multiple sections were used to determine the relative area of dermal layers using ImageJ. Quantitative data are presented as mean ± S.E. of three paw sections each from individual rats (*n* = 3). *P < 0.05, ** *P* < 0.01, and *** *P* < 0.001 compared to the CRG group

To examine the effects of LIUS on the histopathological changes in the inflamed paw by carrageenan, the sections of paw tissue from each group with or without 100 mW/cm^2^ of LIUS were stained by H&E. LIUS stimulation notably inhibited the paw edema formation (Fig. 2). Notably, we have previously shown that the 100 mW/cm^2^ intensity ultrasound significantly decreases water content in the rat brain edema model [15]. Figure 2 shows the histopathological changes in the paw tissue at 6 and 24 h after the injection of carrageenan. Saline-injected rats demonstrated the preservation of typical architecture of paw tissue with collagen fibers and the presence of fibroblasts. Carrageenan-injected paws (CRG) showed a diffused inflammation characterized by interstitial edema at 6 h after the injection. However, the rats treated with LIUS at the intensity of 100 mW/cm^2^ for 10 min (CRG + US) exhibited less interstitial edema than the carrageenan-injected rats, which is consistent with a reduction in the paw volume. On the other hand, carrageenan-injected paws did not show marked differences in the tissue architecture and paw edema at 24 h following the carrageenan injection regardless of LIUS stimulation. In addition, these histological changes were evaluated with relative areas of dermis in paw tissue quantitatively. As shown in Fig. 2b, the average dermis area was enlarged by almost four folds after 6 h following the carrageenan injection. However, LIUS significantly inhibited the increase of the dermis area.

### Effects of LIUS on vascular permeability

**Fig. 3:**
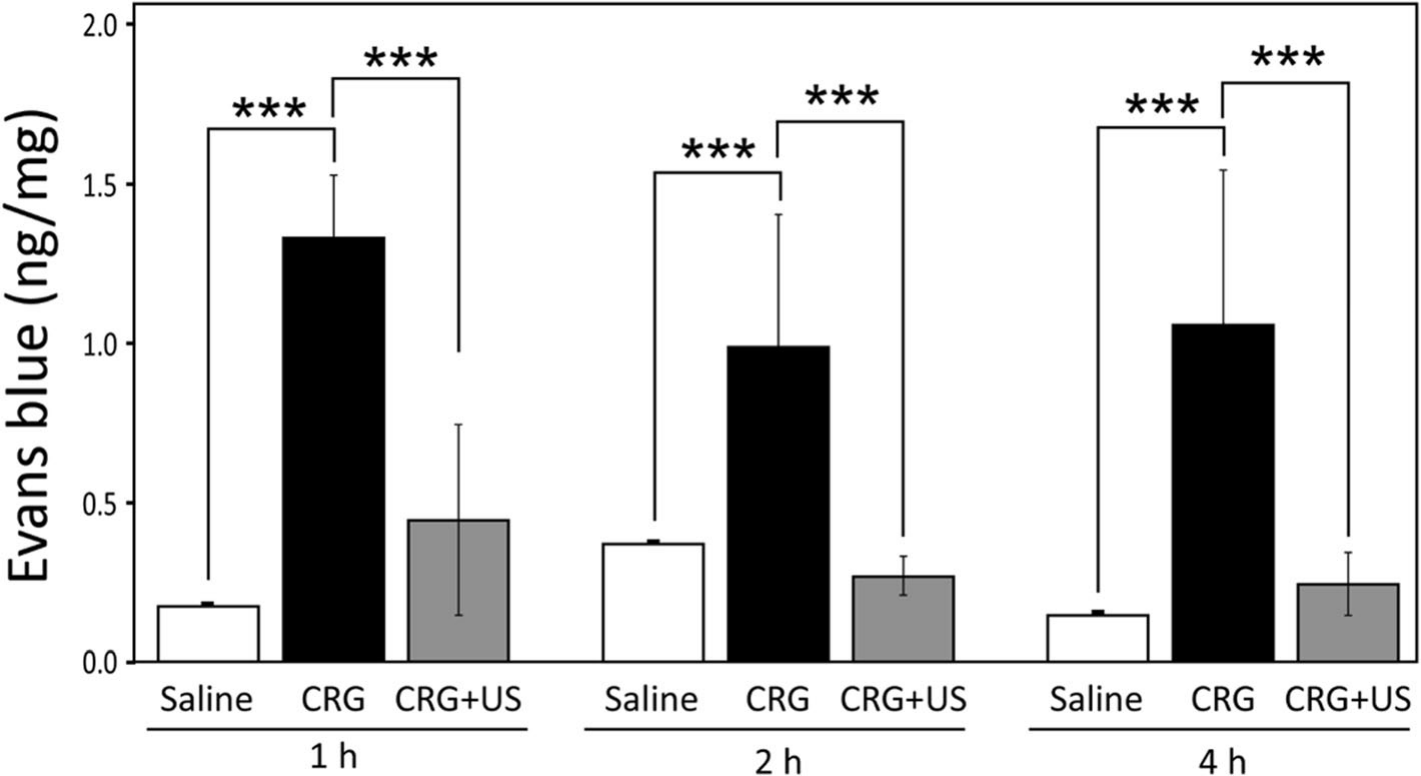
Effect of LIUS stimulation on the vascular permeability. Evans Blue solution was intravenously administrated 30 min before the carrageenan injection with or without LIUS stimulation. Paw tissue was incubated with 10% neutral buffered formalin to extract extravasated Evans Blue. Optical density was measured at 630 nm and the measurements converted into ng dye extravasated per mg tissue. The extent of Evans Blue extravasation was evaluated at 1, 2 and 4 h after treatment in the saline, CRG and CRG + US groups. Data were presented as mean ± S.E. of 3 independent experiments (*n* = 3). *P < 0.05, ** *P* < 0.01, and *** *P* < 0.001 compared to the CRG group

Carrageenan is known to trigger the release of histamine and kinins as mediators of vascular leakage during inflammation. We next tested whether LIUS stimulation can regulate the vascular permeability in the presence of carrageenan. Intravenous injection of Evans Blue was used to evaluate the changes in vascular permeability of the inflamed paw. Evans Blue is a tracer that binds to albumin and is used as an index of plasma protein leakage into tissue [19]. After subplantar injection of carrageenan into the hind paw, the extravasation of Evans Blue was increased in CRG group (1 h, 1.33 ± 0.19; 2 h, 0.99 ± 0.4) compared to saline group (1 h, 0.18 ± 0.01; 2 h, 0.37 ± 0.01) but markedly reduced in CRG + US group (1 h, 0.45 ± 0.29; 2 h, 0.27 ± 0.06), respectively (Fig. 3). These results suggest that LIUS inhibits the increase of vascular permeability by carrageenan in vivo.

### Effects of LIUS on the expression of iNOS and COX-2

**Fig. 4:**
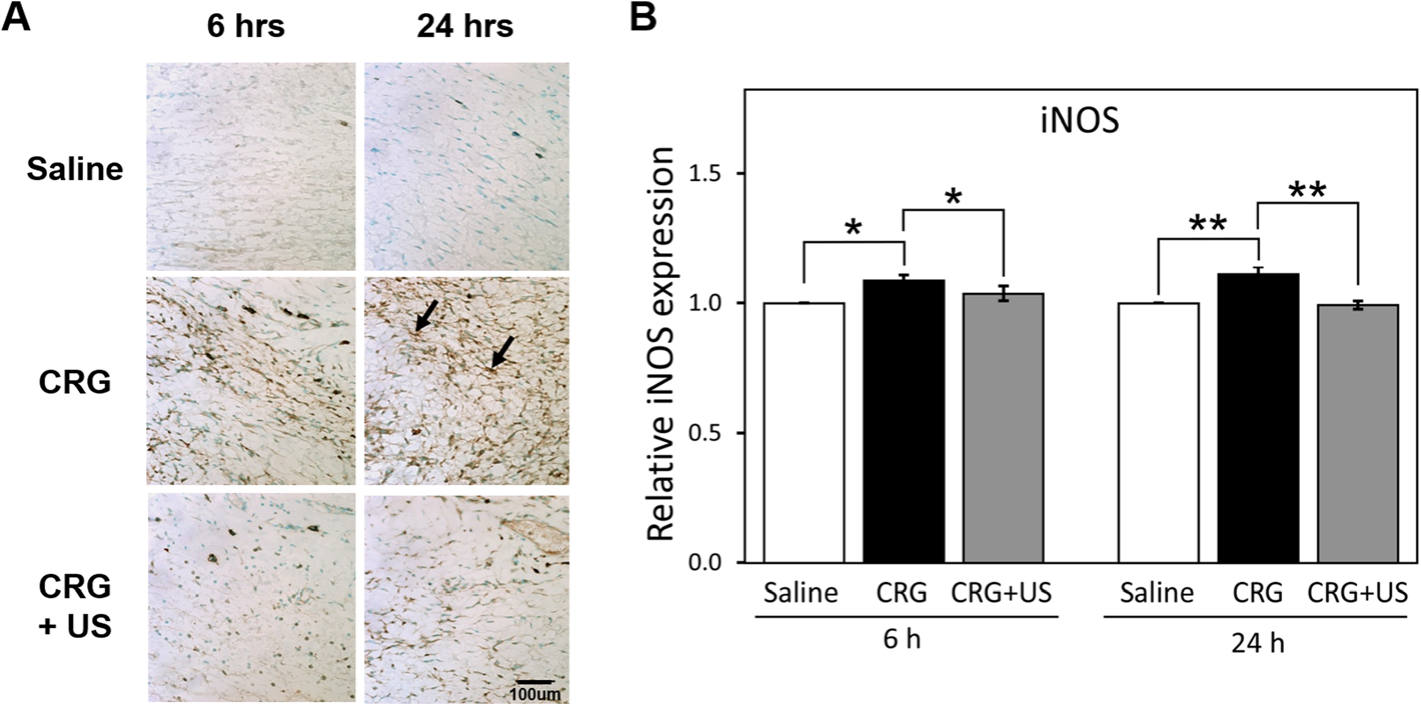
Immunohistochemical staining of iNOS in the paw tissue. **a** Paw tissue sections were immunostained for iNOS at 6 and 24 h in the saline, CRG, and CRG + US groups. Arrows indicate representative iNOS positive cells. **b** The expression levels of iNOS are quantified by ImageJ. The graph shows relative amount of iNOS expression to that of the saline group. Data were presented as mean ± S.E. of 3 independent experiments (*n* = 3). Statistical comparison was conducted on the saline versus CRG groups and the CRG versus CRG + US groups. *P < 0.05, ** *P* < 0.01, and *** *P* < 0.001. Scale bar: 100 μm

**Fig. 5:**
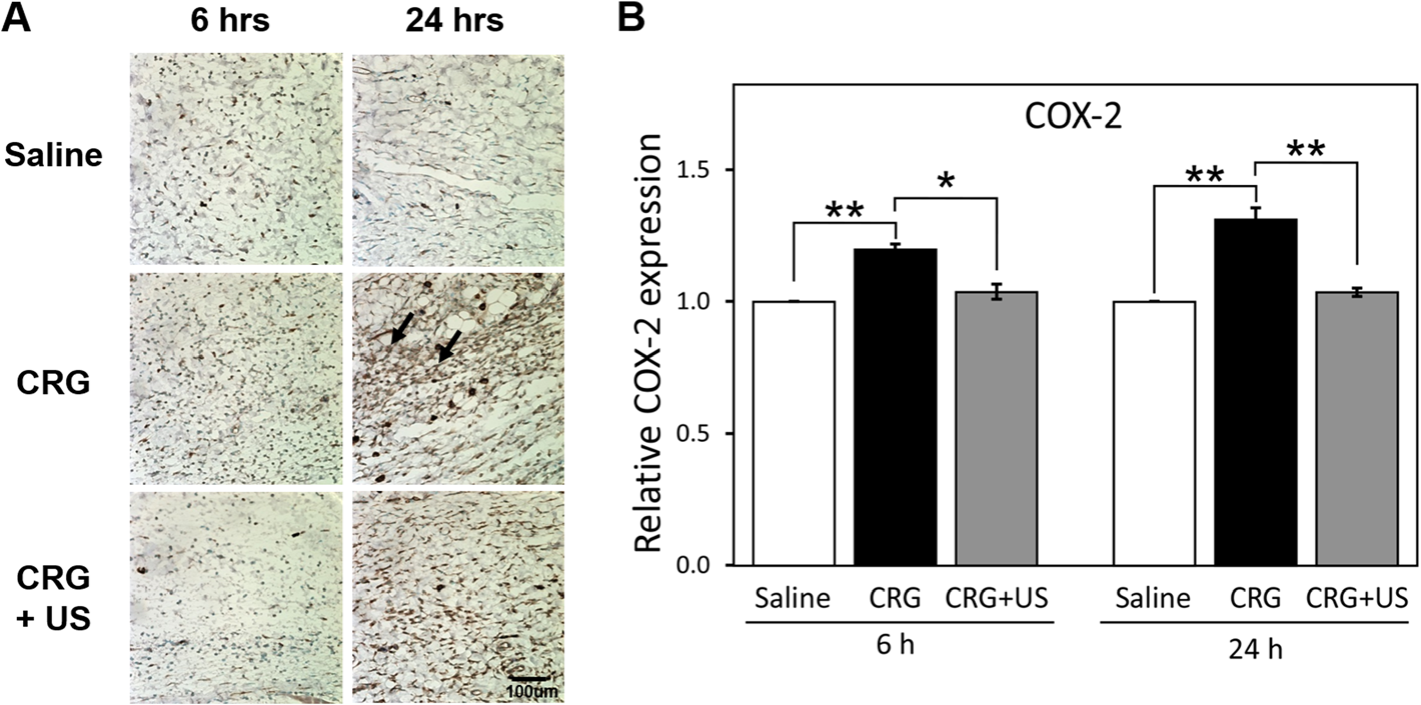
Immunohistochemical staining of COX-2 in the paw tissue. **a** Paw tissue sections were immunostained for COX-2 at 6 and 24 h in the saline, CRG, and CRG + US groups. Arrows indicate representative COX-2 positive cells. **b** The expression levels of COX-2 are quantified by image J. The graph shows mean ± S.E. of 3 independent experiments (*n* = 3). Statistical comparison was conducted on the saline versus CRG groups and the CRG versus CRG + US groups. *P < 0.05, ** *P* < 0.01, and *** *P* < 0.001. Scale bar: 100 μm

The paw tissues were examined immunohistochemically for the presence of iNOS (Fig. 4) and COX-2 (Fig. 5). Immunohistochemical analyses of the paw tissues of saline-injected rats showed no iNOS or COX-2 staining. In contrast, at 6 h after carrageenan injection, specific iNOS immunoreactivity was largely observed and localized to discrete cells within the inflamed paw tissue (Fig. 4a). The expression of COX-2 was also found to be localized to the dermal layer of the inflamed paw skin alone (Fig. 5a). The COX-2 immunoreactivity was much stronger at 24 h rather than at 6 h after carrageenan administration. However, when the inflamed paw was stimulated with LIUS, the iNOS and COX-2 expression was markedly reduced both at 6 h and 24 h compared to the carrageenan alone (Fig. 4b and 5b). These data demonstrate that LIUS stimulation downregulates the inflammatory mediators such as iNOS and COX-2 during the early phase of the inflammatory response.

## Discussion

Carrageenan is well known to induce acute and local inflammations resulting in edema formation [20]. In the carrageenan-induced edema model, histamine, bradykinin, and endothelial-derived nitric oxide (NO) are known as the mediators of permeability in the early phase. These molecules bind to their corresponding receptors and activate specific signaling pathways and induce the development of transient alterations in focal discontinuities between adjacent endothelial cells [21].

Our current study showed that carrageenan injection in rat paws induced edema and decreased vascular permeability within 6 h. Histopathological analyses of the paw tissue at the injected site also exhibited the presence of polymorphonuclear leukocyte at 6 h after the carrageenan injection. When carrageenan-injected rat paws were stimulated with LIUS, a significant reduction in both paw swelling and infiltration of inflammatory cells at the early phase after carrageenan injection were observed. Moreover, a significant decrease in the vascular permeability at least until 2 h in the paw tissue was seen after by LIUS stimulation.

Recent studies have shown that LIUS stimulation decreases edema formation in in vivo and in vitro experiments. In a rat traumatic brain injury model created using the weight-drop method, LIUS stimulation showed a significant decrease in brain water content and the permeability of the blood-brain barrier (BBB) when the brain was immediately stimulated with LIUS after brain injury [11]. However, the delayed stimulation of LIUS did not show any decline in brain water content and BBB permeability. Similarly, we also found that when the inflamed paw was shortly stimulated with LIUS after carrageenan injection, the paw edema and vascular permeability were markedly decreased as compared with the unstimulated control. However, the differences in paw edema and vascular permeability were insignificant when the LIUS stimulation was applied at 2 h after carrageenan injection (data not shown). These results suggest that LIUS may have a protective effect on edema formation and vascular permeability changes by regulating some signal pathways and/or membrane proteins involved in fluid movement from blood vessels into interstitial tissue at the early stage of the pathogenesis.

We also examined the expression levels of iNOS and COX-2, and found that LIUS inhibited the upregulation of both iNOS and COX-2 expressions at the early phase after carrageenan injection. With these results, we assume that LIUS possibly inhibits vascular permeability through downregulation of iNOS, subsequently affecting the COX-2 expression at the early phase of carrageenan-induced inflammation. Downregulation of iNOS and COX-2 expression might be involved in the inhibitory effect of LIUS on paw edema formation. This is probably in line with our recent finding that LIUS stimulation reduced the high glucose level-induced expressions of iNOS and COX-2 in retinal pigment epithelial cells [22].

Therapeutic US can be subdivided into two categories depending on its main biological effects, namely thermal and non-thermal effects [23]. The biological effects of LIUS have been shown to involve the non-thermal effects, including shear stress and cavitation, rather than the thermal effects [24]. These physical forces induce both biomechanical and biochemical changes in cells or tissues. The biological effects of LIUS on a specific cell and tissue can be altered by various LIUS parameters, including frequency, intensity, stimulation time, and the type of waveform, namely pulsed or continuous.

Previous studies also reported the therapeutic effects of LIUS on edema and inflammation, with different experimental conditions in vitro and in vivo. Lim et al. demonstrated that LIUS stimulation can reduce gramicidin D-induced erythrocyte edema with continuous wave US for 10 min at a frequency of 1 MHz and intensities of 30, 70, and 100 mW/cm^2^. The reduction in erythrocyte edema indicated no significant difference with respect to the US intensities used. Yoon et al. showed that LIUS stimulation of brain tissue at 1 MHz and 100 mW/cm^2^ for 5 min inhibited the increase in brain water content and the spectrophotometric absorbance of Evans blue dye after brain injury. Another study also reported the anti-inflammatory effect of LIUS at 200 mW/cm^2^ on complete Freund’s adjuvant (CFA)-induced arthritis synovium in rats [13]. These studies partly agree with our observation of paw volume changes in terms of LIUS parameters. We also used LIUS to stimulate paw tissues at a frequency of 1 MHz and intensities of 50, 100, and 200 mW/cm^2^ for 10 min. All these intensities showed similar effects on the paw edema changes at the early phase. Thus, the US intensity range that we used in the present study may not be a critical factor to reduce the paw edema formation in this experimental condition, at least in part. It is suggested that the LIUS intensity has a wider range from 30 to 200 mW/cm^2^ to induce biological effects in various cells and tissues. For example, Kim et al. reported that LIUS has the potential to treat oxidative damage at intensities from 50 to 200 mW/cm^2^ in retinal pigment epithelial cells [25].

## Conclusions

Our findings suggest that LIUS significantly inhibits paw edema formation and vascular permeability in intraplantar carrageenan-induced paw edema in rats. The inhibitory effect of LIUS on paw edema is likely to be mediated through the downregulation of the iNOS and COX-2 expression during the early phase. Taken together, these results suggest that mechanical stimulation by LIUS could be a potent therapeutic tool for the treatment of inflammatory limb edema. However, our results might not be directly extended to reflect the effectiveness of LIUS for the treatment of systemic edema because the pathological mechanism of local limb edema formation is different from that of systemic edema caused by, for example, nephrotic syndrome and heart failure.

## Materials and methods

### Animals

Male Sprague-Dawley rats (10–12 weeks old) were purchased from Orient Bio Inc., Korea. All animals used in this study were treated in accordance with INHA University-Institutional Animal Care and Use Committee (INHA-IACUC) on their ethical procedures and scientific care.

### Carrageenan-induced paw edema model

The carrageenan-induced edema model was established with a slightly modified method than the one previously published [26]. Briefly, the rats were anesthetized, and given a subplantar injection of 100 μL of 1% (w/v) carrageenan lambda (λ-carrageenan, type IV, Sigma Aldrich, St. Louise, MO, USA) diluted in saline in the left hind foot pad. The animal subjects were divided into three groups as follows. (i) saline: rats were injected with saline alone; (ii) CRG: rats were injected with carrageenan; and (iii) CRG + US: rats were stimulated with LIUS for 10 min on the left hind foot right after carrageenan injection.

### LIUS stimulation

**Fig. 6:**
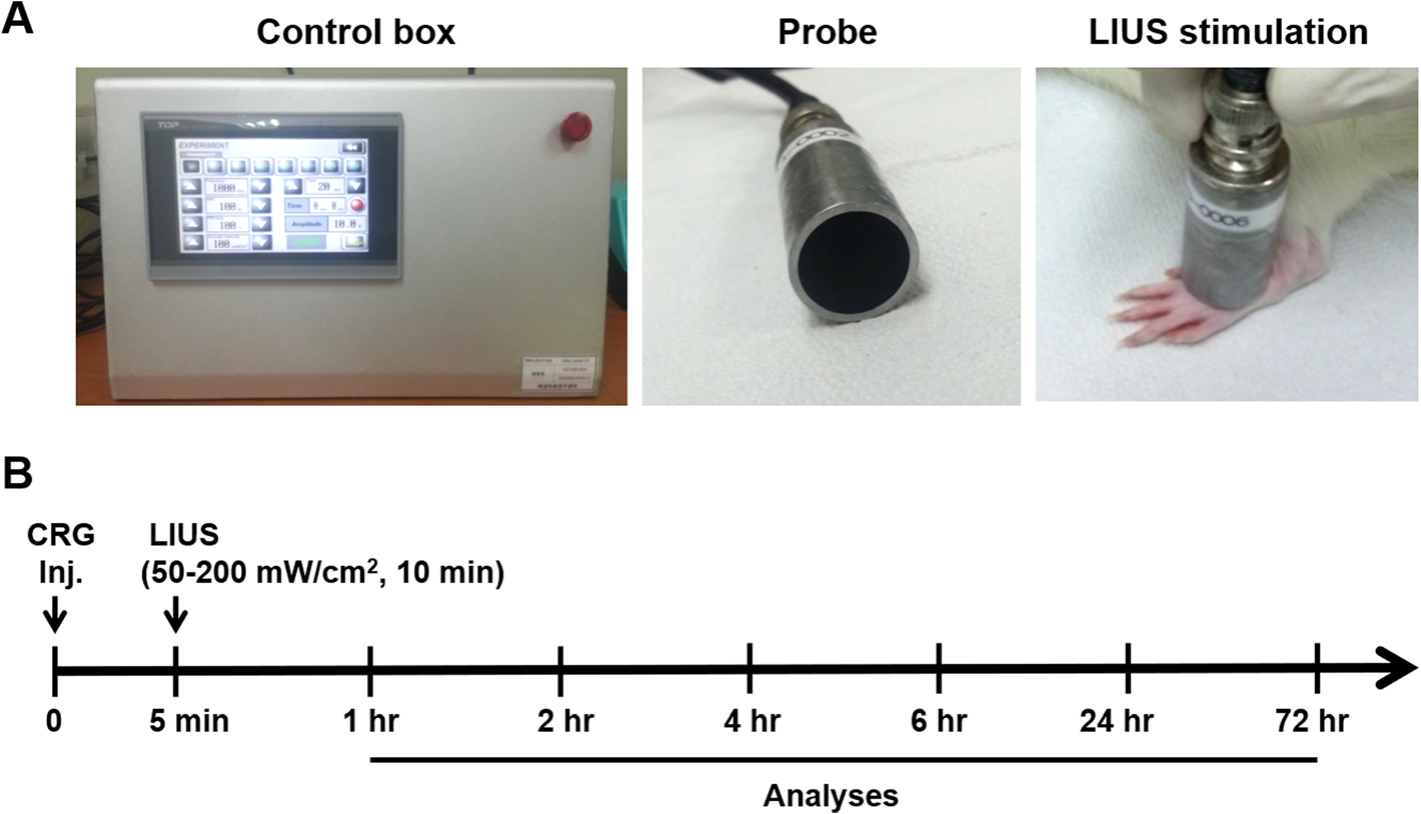
The experimental set-up. **a** A custom-made LIUS generating device (Korust, Korea). The ultrasound generating device allows for adjusting the intensity range from 20 to 1000 mW/cm^2^ and the treatment time through the control box (left panel). The device has two probes with 11 mm in diameter customized for animal experiments (middle panel). A probe was placed on top of the hind paw of a rat for LIUS stimulation (right panel). **b** A schematic diagram showing the study protocol in vivo. The rats were stimulated with LIUS (50–200 mW/cm^2^) for 10 min right after carrageenan injection, and then the paw tissue was analyzed at a given time points from 1 h to 72 h depending on assays

Rats were anesthetized with pentobarbital sodium (60 mg/kg, Hanlim Pharm. Co., Seoul, Korea). For the LIUS stimulation, a custom-made ultrasound generator (Korust Co., Anyang, Korea) were used. The intensity and the treatment time of ultrasound can be controlled in the apparatus (Fig. 6a). LIUS was generated in a continuous-wave mode at a frequency of 1 MHz and intensity of 50, 100, and 200 mW/cm^2^ for 10 min. LIUS stimulation was applied on the left hind paw following the carrageenan injection (Fig. 6a and b). The saline and CRG groups were also placed on the transducer probes without applying the ultrasound.

### Assessment of paw edema

Paw volumes were measured at times 0, 2, 6, 24, and 72 h after carrageenan injection using a water plethysmometer according to the manufacturer’s instructions (Ugo Basil, Italy). Briefly, measurements were taken for the injected and contralateral hind paws. Hind paw was inserted into the water plethysmometer up to the determined ankle line, then recorded the volume changes before and after the injection. Rats were euthanized by carbon dioxide inhalation. The soft paws were flash frozen in liquid nitrogen and kept at − 70 °C for other assays.

### Histopathology and immunohistochemistry

For histopathological examination, biopsies of paws were taken 6 or 24 h following the carrageenan injection. Rats under deep anesthesia induced by pentobarbital sodium (100 mg/kg) were initially perfused intracardially with 200 mL of cold PBS containing 1% sodium nitrite and heparin (0.2 U/mL), and then with 4% paraformaldehyde in 200 mL of PBS (pH 7.4). The paw tissues were harvested and further fixed in 10% neutral buffered formalin. The paws were bisected longitudinally, placed in embedding cassettes, embedded in paraffin, and then cut into 4 μm sections. The sections around the injected site were stained with hematoxylin and eosin (H&E) for histopathological observations. The multiple sections were used to determine the relative area of dermal layers in paw tissue using ImageJ software. For immunohistochemistry, endogenous peroxidase in the sections was quenched with 10% H_2_O_2_ in 60% methanol for 30 min following deparaffinization. The sections were incubated overnight at 4 °C with anti-iNOS (1:500 dilution; Abcam, Cambridge, MA, USA) or anti-COX-2 (1:500 dilution; Abcam, Cambridge, MA, USA) antibodies. Specific labeling was detected with a biotin-conjugated goat anti-rabbit IgG and an avidin-biotin peroxidase complex (DBA; Vector, Milan, Italy). Finally, the sections were reacted with 3, 3′-diaminobenzidine tetrahydrochloride (DAB) for 30 min. For quantitative analyses, immunohistochemically stained sections were visualized and images were captured. The intensities of the immuno-labels were quantified using ImageJ software.

### Measurement of extravasation of Evans blue

Evans Blue solution (50 mg/kg of 50 mg/mL solution) was injected intravenously immediately before the subplantar injection of carrageenan. The rats were sacrificed after 1, 2, and 4 h, and the inflamed tissues were taken by the punch biopsy tool (5 mm diameter) on the site of injection. The tissues obtained were shaken gently in 10% neutral buffered formalin for 72 h at 60 °C to allow Evans Blue extraction. Each sample was then filtered and the absorbance of the filtrate was assessed at 630 nm using UV-spectrophotometer. The extracted amount of dye was calculated by extrapolating with standard curve prepared with different concentrations of Evans Blue solution. The data were converted into ng of dye extravasated per mg of tissue.

### Statistical analysis

The quantitative data were presented as mean values ± standard deviations (SD). The statistical significance was analyzed between groups by the non-parametric Kruskal-Wallis test. *P* values of less than 0.05 were considered significant.

## Data Availability

The datasets used and/or analyzed during the current study are available from the corresponding author on reasonable request.

## References

[1] Posadas I, Bucci M, Roviezzo F, Rossi A, Parente L, Sautebin L (2004). Carrageenan-induced mouse paw oedema is biphasic, age-weight dependent and displays differential nitric oxide cyclooxygenase-2 expression. Br J Pharmacol.

[2] Vane JR, Botting RM (1995). New insights into the mode of action of anti-inflammatory drugs. Inflamm Res.

[3] Wallace JL, Reuter B, Cicala C, McKnight W, Grisham MB, Cirino G (1994). Novel nonsterodial anti-inflammatory drug derivatives with markedly reduced ulcerogenic properties in the rat. Gastroenterology.

[4] Moncada S, Higgs A (1993). The L-arginine-nitric oxide pathway. N Engl J Med.

[5] Davidge ST, Baker PN, McLaughlin MK, Roberts JM (1995). Nitric Oxide Produced by Endothelial Cells Increases Production of Eicosanoids Through Activation of Prostaglandin H Synthase. Circ Res.

[6] Sautebin L, Ialenti A, Ianaro A, Di Rosa M (1995). Modulation by nitric oxide of prostaglandin biosynthesis in the rat. British J Pharmacol.

[7] Naito K, Watari T, Muta T, Furuhata A, Iwase H, Igarashi M (2010). Low-intensity pulsed ultrasound (LIPUS) increases the articular cartilage type II collagen in a rat osteoarthritis model. J Orthop Res.

[8] Paliwal S, Mitragotri S (2008). Therapeutic opportunities in biological responses of ultrasound. Ultrasonics.

[9] Saturnino-Oliveira J, Tomaz MA, Fonseca TF, Gaban GA, Monteiro-Machado M, Strauch MA (2012). Pulsed ultrasound therapy accelerates the recovery of skeletal muscle damage induced by *Bothrops jararacussu* venom. Braz J Med Biol Res.

[10] Young S, Hampton S, Tadej M (2011). Study to evaluate the effect of low-intensity pulsed electrical currents on levels of oedema in chronic non-healing wounds. J Wound Care.

[11] Yoon SH, Kwon SK, Park SR, Min BH (2012). Effect of ultrasound treatment on brain edema in a traumatic brain injury model with the weight drop method. Pediatr Neurosurg.

[12] Park SR, Jang KW, Park SH, Cho HS, Jin CZ, Choi MJ (2005). The effect of sonication on simulated osteoarthritis. Part I: effects of 1 MHz ultrasound on uptake of hyaluronan into the rabbit synovium. Ultrasound Med Biol.

[13] Chung JI, Barua S, Choi BH, Min BH, Han HC, Baik EJ (2012). Anti-inflammatory effect of low intensity ultrasound (LIUS) on complete Freund’s adjuvant-induced arthritis synovium. Osteoarthritis Cartilage.

[14] Lim MH, Seo AR, Kim J, Min BH, Baik EJ, Park SR (2014). Effects of low-intensity ultrasound on gramicidin D-induced erythrocyte edema. J Ultrasound Med.

[15] Karmacharya MB, Kim KH, Kim SY, Chung J, Min B-H, Park SR (2015). Low intensity ultrasound inhibits brain oedema formation in rats: potential action on AQP4 membrane localization. Neuropathol Appl Neurobiol.

[16] Bucci M, Roviezzo F, Posadas I, Yu J, Parente L, Sessa WC (2005). Endothelial nitric oxide synthase activation is critical for vascular leakage during acute inflammation in vivo. Proc Natl Acad Sci U S A.

[17] Nantel F, Denis D, Gordon R, Northey A, Cirino M, Metters KM (1999). Distribution and regulation of cyclooxygenase-2 in carrageenan-induced inflammation. British J Pharmacol.

[18] Handy RLC, Moore PK (1998). A comparison of the effects of L-NAME, 7-NI and L-NIL on carrageenan-induced hindpaw oedema and NOS activity. British J Pharmacol.

[19] Yao L, Xue X, Yu P, Ni Y, Chen F (2018). Evans Blue Dye: A Revisit of Its Applications in Biomedicine. Contrast Media Mol Imaging.

[20] Winter CA, Risley EA, Nuss GW (1962). Carrageenin-induced edema in hind paw of the rat as an assay for antiiflammatory drugs. Proc Soc Exp Biol Med Soc Exp Biol Med.

[21] Di Lorenzo A, Fernandez-Hernando C, Cirino G, Sessa WC (2009). Akt1 is critical for acute inflammation and histamine-mediated vascular leakage. Proc Natl Acad Sci U S A.

[22] Karmacharya MB, Hada B, Park SR, Choi BH (2018). Low-Intensity Ultrasound Reduces High Glucose-Induced Nitric Oxide Generation in Retinal Pigment Epithelial Cells. Ultrasound Med Biol.

[23] Dalecki D (2004). Mechanical bioeffects of ultrasound. Annu Rev Biomed Eng.

[24] Feril LB, Kondo T (2004). Biological effects of low intensity ultrasound: the mechanism involved, and its implications on therapy and on biosafety of ultrasound. J Radiat Res.

[25] Kim NK, Kim CY, Choi MJ, Park SR, Choi BH (2015). Effects of low-intensity ultrasound on oxidative damage in retinal pigment epithelial cells in vitro. Ultrasound Med Biol.

[26] Miyazaki T, Sakamoto Y, Yamashita T, Ohmoto K, Fujiki H (2011). Anti-edematous effects of tolvaptan in experimental rodent models. Cardiovasc Drugs Ther.

